# Mine Tailings as a Sustainable Filler for Asphalt Binder–Mastics: A Review

**DOI:** 10.3390/ma18214892

**Published:** 2025-10-25

**Authors:** Daniel O. Oguntayo, Nuha S. Mashaan, Sanjay K. Shukla

**Affiliations:** 1 School of Engineering, Edith Cowan University, Joondalup, WA 6027, Australia; 2 Founding Geotechnical and Geoenvironmental Engineering Research Group Leader, School of Engineering, Edith Cowan University, Joondalup, WA 6027, Australia; s.shukla@ecu.edu.au

**Keywords:** mining waste, tailings, asphalt binder–mastics, physical properties, rheological properties, sustainability, circular economy

## Abstract

Sustainability has become a primary focus on a global scale. However, there is a threat to sustainable development due to the substantial generation of waste from urbanization and industrialization. Particularly, the accumulation of mine tailings is a problem, an industrial waste by-product from the mining sector. To support sustainable development and promote circular economy, it is imperative to utilize the materials in the production of asphalt mastics. This review examines the performance of mining waste-modified asphalt binder–mastic to create an idea of what tailings might be used for in subsequent studies. This review contributes to knowledge by consolidating research efforts on mine tailing-modified asphalt binder–mastics, tailings characteristics for intended usage and the possibility of improved blends. The findings show that the mine tailings improve the physical and rheological properties of asphalt binder–mastic compared to conventional fillers, thereby contributing to more durable and sustainable pavements. Also, the mine tailings are environmentally friendly and economically viable, making them a potential alternative filler to substitute the traditional fillers. The review concluded by outlining the limitations of using tailings and suggested future directions for large-scale utilization of tailings in asphalt binder–mastics.

## 1. Introduction

Mining is the process of extracting materials from the earth to recover one or more valuable minerals. However, this process also generates mining waste—unwanted by-products in solid, liquid, or gaseous form—that currently have no economic value. Despite this, mining plays a critical role in modern society, especially in industries such as electronics and power generation [1,2]. Therefore, continued progress requires increased mining activity. However, this also leads to a rise in mining waste. Currently, an estimated 65 to 80 billion tons of mining waste are generated each year [3,4]. An estimated portion of about 10 to 15 billion tons out of the mining waste generated annually are tailings, while the remaining amount is waste rock, overburden soil, and slag [3,4]. Mine tailings are pulverized rock that remains after the valuable metal-bearing minerals have been extracted in separation processes [5]. Their disposal is challenging because of their heavy metals in their chemical composition and their nature. For safe and effective handling of these tailings, they are stored in a special design dam called tailings dam. However, this also is detrimental to sustainability and consequently results in numerous environmental issues [6]. Thus, for supporting sustainable development in mining activities, an in-depth policy for mine tailings management is vital.

The key element in most road infrastructural assets is asphalt mixtures, making them the dominant road construction material [7]. Around the word, approximately 90% of the road network is made of asphalt, with about 275 million tons of asphalt materials used for its construction annually [8]. Specifically in Western Australia, this road network comprises about 170,000 km of secondary and local roads with more than 18,000 km of main roads and highways. With this vast network of roads, a robust commitment to low-cost pavement construction using recycled materials is necessary [9,10,11], because its construction as the pavement choice employs vast amounts of raw materials and energy. The key constituent in asphalt concrete that is crucial to the design, construction, and maintenance is the asphalt mastic. How well an asphalt mixture performs is dependent on the characteristics of asphalt mastic and might even be more decisive than those of asphalt binder [12]. It controls viscoelasticity performance and is vital to the adhesion in asphalt mixtures [13,14]. Asphalt mastic is a composite material comprising mineral filler particles and asphalt as the binder. The performance of asphalt mastic is significantly influenced by mineral fillers, indicating that fillers are an essential constituent of asphalt mastic. To influence mastic behavior, fillers amend the characteristics of the asphalt binder, thereby enhancing their performance and durability [15,16,17]. This improvement is due to their stiffening capacity, consequently modifying their properties. Conventional materials such as limestone powder are generally employed as fillers in asphalt mastics, though they are not renewable. Then, to enhance the performance of asphalt mastics in the face of changing climate and preserve the non-renewable natural resource deposit, it is imperative to diversify the materials used in the production of asphalt mastics.

Over the years, the use of waste materials has been studied and projected as viable material to replace the conventional materials in asphalt mixtures [18,19,20,21]. In particular, the utilization of mine tailings from mining activities has recently been known to hold potential as emerging filler in asphalt binder–mastics. In [12], the effects of iron tailings (IT) as filler on the fatigue performance of asphalt binder–mastic were investigated and compared with conventional limestone filler. It was reported that the asphalt binder–mastics fatigue performance meets the requirements, indicating that the effectiveness of ITs as a substitute for limestone in asphalt pavement application provides a new perspective on sustainable mine tailings utilization and consequently creates huge social, economic, environmental, and performance benefits. Ref. [22] explored the application of molybdenum tailings (MT) on the rheological and environmental performance of asphalt binder–mastic. They showed that MT increased viscosity, complex modulus, creep stiffness, and rutting factor of asphalt binder–mastic and decreased the creep rate, phase angle, and viscosity temperature sensitivity index, demonstrating that replacing limestone with MT will result in improved asphalt pavement performance and reduce environmental pollution caused by MT disposal. The rheological performance of asphalt binder–mastic-modified magnetite tailings was investigated by [23]. They reported an increase in the asphalt binder–mastic stiffness and elastic behavior at high temperature and a decrease in the loading time and temperature susceptibility, signifying magnetite tailings in enhancing the performance of asphalt pavement and representing a sustainable approach to recycling this material.

Based on these studies, asphalt binder–mastics involving mine tailings can be very beneficial in enhancing performance of asphalt pavement, making them receive much attention. Hence, there is need to explore the existing research on mine tailings-modified asphalt binder–mastics to provide foundation for future studies. This study therefore conducts a review on the research efforts on mine tailing as a sustainable filler in asphalt binder–mastic, to advance understanding and inform future research.

## 2. Significance of the Study

Several studies have focused on recycling mine tailings as raw materials in the field of civil engineering. Obviously, while using mine tailings in broader civil engineering applications have been well documented, their application in asphalt mixtures is a relatively emerging field. There is a growing body of research that supports the potential benefits of these materials in asphalt mixtures, yet comprehensive reviews focused on this application are limited. This review aims to fill that gap by systematically analyzing the latest research, identifying trends, and assessing the feasibility of using diverse mine tailings in asphalt binder–mastic. Furthermore, prominent reviews on mining wastes in asphalt mixtures has been conducted and has offered a comprehensive overview of various mining wastes (slags, waste rocks, and tailings) and their utilization in asphalt mixtures. This review differs by specifically focusing on tailings as a constituent within the asphalt binder–mastic, rather than their use as aggregates or fillers in the overall mixture, providing a focused investigation into a particular aspect of mining waste integration in pavements. By exploring the under-researched area of tailings in asphalt binder–mastics, this review provides research efforts of the role of tailings in asphalt binder–mastics, offering valuable insights for future research and applications.

A comprehensive search of major databases, including PubMed, Scopus, Web of Science, and Google Scholar were conducted using strings such as “tailings”, “asphalt binder–mastic” with Boolean operators “AND/OR”. Studies published up to 2025 were included based on relevance to the topic, publication in peer-reviewed journals, and availability of full-text articles. Exclusion criteria included non-English language publications and studies with methodological limitations.

## 3. Mine Tailings Management

Mine tailings are mining waste materials and they represent approximately 10–20% of by-products generated by the mining industry [4,24]. Mine tailings are pulverized rock that remains after the valuable metal-bearing minerals have been extracted in separation processes [5]. They are mainly composed of finely ground sand to silt-sized rock particles, water, and processing reagents used to extract valuable minerals from the ore [25,26]. Their disposal challenging because of their heavy metals in their chemical composition and their nature [1]. In view of the significant negative environmental effects of tailings dumping into open lands and water bodies, they are dumped in a special design dam called tailings dam. However, immense expanses of land are required for the storage of the vast amounts of tailings, which not only reduces the availability of arable land but also brings significant economic costs [27]. Several disastrous tailing dam failures have also occurred [28,29,30]. For instance, in 11th September 2022, there was a catastrophic failure of Jagersfontein mine’s tailings dam in South Africa, resulting in the abrupt and wild release of over 6 million cubic meters of toxic liquid sludge, damaging about 200 neighboring houses and approximately 1600 ha of arable land [31,32]. To reduce the potential adverse environmental impacts and long-term storage risk, it is highly desirable to adopt effective and efficient methods to utilize in large scale as sustainable alternative resources [33]. One of such sustainable alternatives is the utilization of mine tailings as road construction material. This will help curb the adverse environmental impact associated with tailings management, aid in the transition to circular economy, preserve the non-renewable natural resource deposit used as road construction materials, and, as well, enhance the performance of asphalt pavement.

## 4. Mine Tailings Characteristics as Fillers in Asphalt Binder–Mastics

The characteristics of tailings are dependent on the source of ore and the ore extraction process. The physical and chemical properties of selected mine tailings in asphalt mixtures are depicted in Table 1 and Table 2 and selected filler properties are shown in Table 3. Typically, SiO_2_, Al_2_O_3_, and Fe_2_O_3_ are abundant in mineral wastes from tailings, indicating that they are pozzolanic in nature, and hence making them desirable options for usage as construction materials. SiO₂ often causes weak adhesion but provides filler strength, while alumina (Al₂O₃) and iron oxide (Fe₂O₃) generally improve adhesion with binder [34,35]. Tailings contain a moderate proportion of CaO, which is needed for its reactivity with bitumen. This unique composition of tailings can improve several properties of asphalt binder–mastics for enhanced performance in asphalt applications. Also, it can be seen that the density of the tailings is relative between 2 and 3.

Furthermore, it can be deduced that the physical and chemical properties of the tailings are comparable to the existing fillers utilized in asphalt mastics as shown in Table 3. This comparison offers mine tailings to be of potential use in asphalt mixtures as an alternative source of filler for sustainable asphalt mixture production. In addition, physical properties and chemical composition of the tailings will play a pivotal part in the performance of asphalt binder–mastics [36]. Nonetheless, it is critical to determine the properties of tailing to be used for this purpose. The type, source, and processing method of the mineral ore determines the tailings’ characteristics since different minerals coexist with various compounds, including silicon and iron [37]. For example, in the study conducted by [38], phosphate mine wastes (PMWs) from difference sources were utilized. The PMWs have different properties but they are in the range of being compatible as filler materials in asphalt mixtures.

**Table 1:** Physical properties of mine tailings.

Mine Tailings	Density (g/cm^3^)	Water Absorption (%)	Hydrophilic Coefficient (g/cm^3^)	Reference
Tungsten tailings	2.89	0.18	-	[39]
Phosphate mine waste	2.455	9.19	-	[38]
Iron tailings	2.95	1.2	-	[12]
Molybdenum tailings	2.64	0.24	0.84	[22]
Copper tailing	2.87	0.3	0.81	[40]
Coal gangue	2.27	-	0.87	
Graphite tailings	2.942	-	-	[41]
Gold tailings	2.75	-	-	[42]
Red mud	3.12	-	0.85	[43]
Manganese tailings	2.95	-	-	[44]

**Table 2:** Chemical composition of mine tailings.

Mine Tailings	Oxides									Ref.
	Al_2_O_3_	CaO	Fe_2_O_3_	MgO	SO_3_	SiO_2_	P_2_O_5_	MnO	K_2_O	
Tungsten tailings	18.39	-	11.85	-	10.94	44.83	-	-	3.62	[39]
Manganese tailings	34.10	0.11	7.33	-	0.40	46.95	0.17	14.95	0.98	[44]
Phosphate Mine waste	1.38	37.47	0.28	0.29	1.72	18.09	2.86	-		[38]
Iron tailings	8.06	4.56	9.52	5.28		66.70	0.43		2.53	[15]
Molybdenum tailings	11.472	3.362	1.853	-	-	71.842	-	0.047	7.32	[22]
Copper tailing	21.19	6.75	6.63	1.47	3.343	49.24	-	1.47	9.02	[40]
Coal gangue	46.11	0.29	0.56	0.10	0.01	50.42	0.51	-	0.23	[45]
Graphite tailings	2.28	0	55.30	-	-	23.52	-	-	-	[41]
Gold tailings	14.76	5.92	13.04	2.40	2.76	41.08	-	2.02	10.79	[42]
Red mud	32.61	2.21	20.65		0.69	27.64	1.77	0.019	0.018	[43]

**Table 3:** Selected commonly used fillers in asphalt mastics.

Properties	Limestone Powder	Cement	Fly Ash	Steel Slag	Lime	Granite Dust	Rice Husk Ash	Bottom Ash
Density (g/cm^3^)	2.67	3.15	2.274	3.58	2.19	2.690	2.21	2.7
CaO	82.828	69.39	0.46	46.29	64.67	2.877	2.89	6
SiO_2_	5.843	22.55	61.44	15.778	11.74	63.87	74.89	50
Al_2_O_3_	4.15	5.02	28.31	1.362	8.68	16.854	1.06	28
Fe_2_O_3_	0.573	2.68	6.87	23.179	2.47	3.116	1.33	12
MgO	4.786	3.69	-	4.17	2.08	0.692	1.96	2
K_2_O	0.351	-	0.37	-	2.35	5.252	6.09	-
Na_2_O	-	-	-	-	1.71	6.023	-	-
Other	1.469	-	0.40	5.634	6.3	1.316	7.26	2
Ref.	[46]	[47]	[48]	[49]	[50]	[46]	[51]	[52]

## 5. Influence of Mine Tailings on the Performance of Asphalt Binder–Mastics

Mine tailings as recycled materials in asphalt mixtures have gained a lot of interest lately. Its utilization in asphalt mixtures as a constituent ranges from as an aggregate to filler materials. The increased performance of asphalt mixtures with mine tailings reflects the increasing attention in the subject. Principally, the attention of mine tailings as a potential filler material in asphalt binder–mastics has attracted a lot of attention because of its abundance, pozzolanic characteristics, low-cost, and potential savings in the utilization of non-renewable resources [44,53,54]. The effects of various mine tailings in asphalt binder–mastics are showed in Table 4. According to the Table, the usage of mine tailings has brought about improved performance of the asphalt mastics. Mining tailings contains a high content of transition metal oxides, which is responsible for the improvement in mastic properties [44]. Hence, the use of mining tailings-based fillers for the production of asphalt binder–mastic could potentially result in improved mastic performance and prolonged lifecycle in addition to being fundamental for circular economy and environmental remediation [44,54]. Mine tailings modified mastic-specific performance in terms of physical, rheological, and moisture resistance, and their interaction capacity with asphalt is discussed in detail in the subsequent section.

### 5.1. Physical Properties of Mine Tailings-Modified Asphalt Binder–Mastics

Conventional bitumen tests like softening point, ductility, penetration, and penetration index are often used as criteria for physical characterization asphalt and asphalt mastics [57,59,60]. The indicator of the viscosity of asphalt and the temperature point at which the phase change starts to occur is the softening point. In the study conducted by [40] on the influence of recycled copper tailings (CTs) as a replacement to limestone filler in asphalt binder–mastic, it was revealed that the softening point increased with the increase CTs content. The improvement in softening point of CT asphalt mastic is valuable because the bitumen is less prone to rutting with a high softening point. Furthermore, at the same filler concentrations, the CT asphalt mastics has an improved softening point compared with limestone–asphalt mastics. In [57], red mud was used as an alternative filler to limestone at a filler/binder ratio of 1.1, and it was found that red mud-modified asphalt mastic resulted in a 14.64% improvement in the asphalt mastic softening point.

To characterize the stickiness of bitumen or asphalt mastic, ductility is a generally used parameter. It has been reported that mine tailings have an adverse effect on the ductility of asphalt mastic [40,57]; this decrease is due to the reduced proportion of structural asphalt, which can be compensated for using a low filler/binder [40] or using hybrid additives [57]. The penetration test is used for describing the consistency of asphalt mastic. As a general rule, it can be said that a softer asphalt mastic means a greater penetration, i.e., a lesser consistency. The higher the consistence of the asphalt mastic at a higher temperature, all the better, as at the same time, it means an increased temperature at the optimum construction consistence level. According to the study conducted by [40], copper tailings asphalt mastics outperform mastics with limestone powder resulting in mastic with high-temperature performance. The larger specific area of the tailings particles is attributed to this better stiffening effect, provided by [61]. Conversely, ref. [57] reported a negative effect of red mud on mastic penetration value. This could be due to the properties and high dosage of filler/binder ratio utilized in their study. In terms of the penetration index (PI) (calculated using Equation (1)), mine tailings improved the PI of the mastic, implying an adverse effect on the mastic low-temperature behavior.

(1) $$PI=\frac{1952-500\log\left({Pen}_{25}\right)-20SP}{50\log\left({Pen}_{25}\right)-SP-120}$$

where SP is the softening point (°C) and *Pen*_25_ is the penetration at 25 °C (0.1 mm).

**Figure 1:**
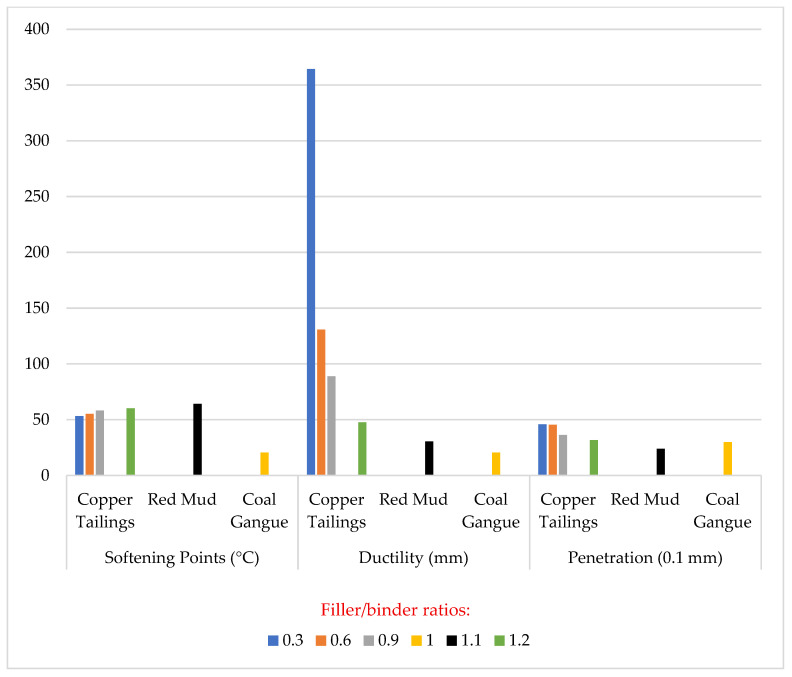
Conventional properties of tailings-modified asphalt mastic [40,57,62].

The summary of the conventional properties of the tailings-modified asphalt mastics is shown in Figure 1. It was observed that as filler/binder ratio increases, there is a decrease in the ductility and penetration with a significant increase in the softening point. Hence, it can be inferred that mine tailings can significantly improve the physical performance of the asphalt mastic. Also, as reported by various studies, tailings-modified mastic exhibits superior conventional properties, which is attributed to the improved stiffening effect provided by a greater specific area of the tailing particles.

### 5.2. Rheological Performance of Mine Tailings-Modified Asphalt Mastics

**Figure 2:**
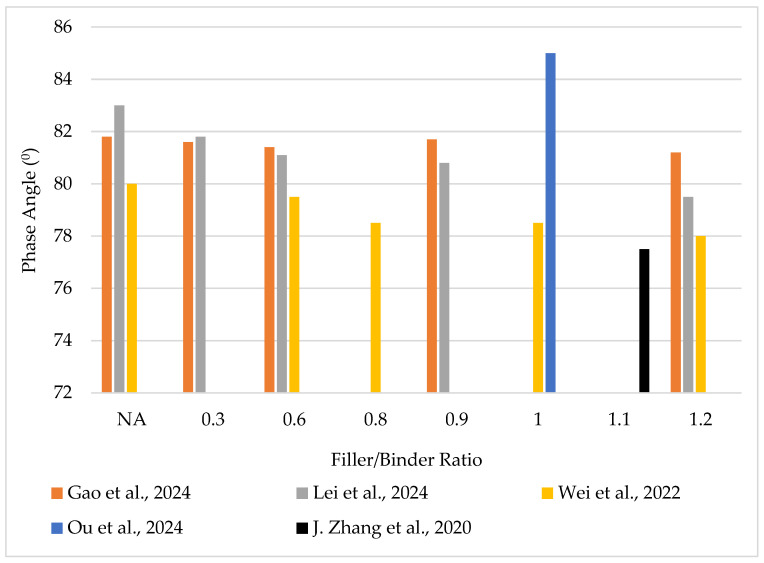
Phase angle of tailings-modified asphalt binder–mastic from different researchers [22,40,55,57,63].

**Figure 3:**
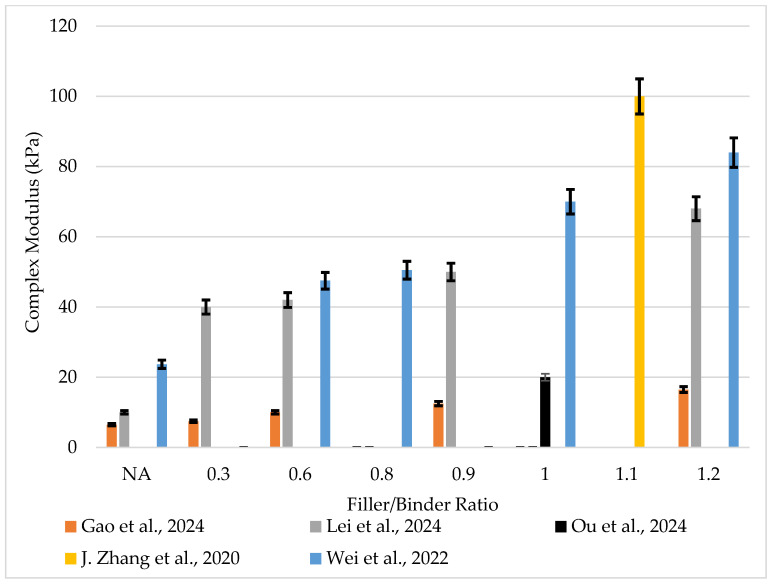
Complex modulus of tailings-modified asphalt binder–mastic from different researchers [22,40,55,57,63].

Asphalt mastic exhibits viscoelastic properties. It behaves as a liquid and a solid under different temperature environments. To understand its performance under load and environmental conditions, its rheological properties must be characterized. It has been generally reported that the mine tailings enhanced the rheological performance of mastics as depicted in Figure 2 and Figure 3.

The viability of using molybdenum tailings (MTs) in asphalt mastic rather than limestone filler (LF) was conducted by [23]. Using filler/asphalt (F/A) ratios of 0.3 to 1.2 with increments of 0.3 to prepare the mastic, the rheological characteristics of the mastics were investigated using a dynamic shear rheometer test. According to their results, the MTs-modified the asphalt binder–mastics and enhanced asphalt’s viscosity, complex modulus, and rutting factor, indicating MTs as a promising filler to lessen environmental pollution by replacing LF in asphalt mastics. In [24], assessment of magnetite tailings from different mining sites for use as filler in asphalt binder–mastics was carried out. The rheology of the magnetite tailings-modified asphalt mastics compared to mastics based on natural limestone filler was examined. The results revealed that with lowering the mastic’s susceptibility to temperature and loading duration, the viscoelastic qualities of the mastics were enhanced. Furthermore, there was an improvement in the elastic and stiffness behavior of the mastic at high temperatures, suggesting that magnetite is suitable as filler in asphalt mastics.

**Figure 4:**
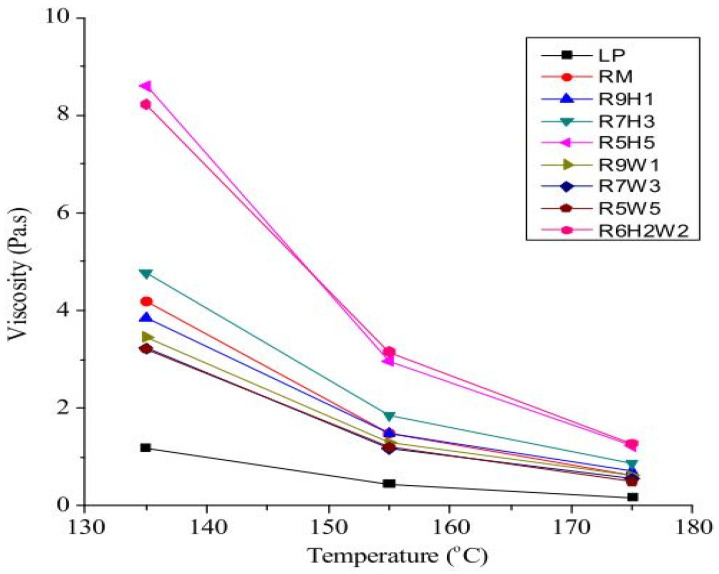
Viscosity of red mud-modified asphalt binder–mastic [57].

In [13], the influence of iron tailings (ITs) fillers in asphalt binder–mastic using 70# bitumen was examined. Using the filler/binder ratio of 1.0 and dynamic shear rheometer (DSR), it was revealed that the performance of ITs-modified asphalt mastic meets the requirements as mineral fillers in asphalt mixtures and has great economic and environmental effectiveness. Ref. [56] also reported that the inclusion of IT at both the same filler concentration and temperature substantially results in higher viscosity than mastic with limestone, indicating that improved mastic viscosity by IT is better than limestone. In the study conducted by [58], red mud was used to replace limestone powder, and they reported a nearly four times increase in the viscosity of the mastic, as shown in Figure 4. They attributed this increase in the viscosity to the larger pore volume and relatively smaller particle size as well as specific surface area of the red mud.

Based on the results mentioned above, the addition of mine tailings resulted in enhanced rheological performance of asphalt mastic. This improvement may be due to the mineralogical composition and surface morphology of tailings compared to the limestone powder of asphalt mastic [58].

### 5.3. Moisture Performance of Mine Tailings-Modified Asphalt Binder–Mastics

The primary type of deterioration in asphalt pavement is the moisture damage [64,65]. Moisture susceptibility is a significant property which is closely associated with the durability, structural stability, and serviceability of asphalt mixtures. Moisture sensitivity stripping, more commonly known as stripping, refers to the process of removing bitumen coating from the surface of an aggregate. This occurs at the point at which initial adhesion bonds between the asphalt mastics and the aggregate surface are broken and is also a result of cohesion failures in the asphalt cement [66,67].

**Figure 5:**
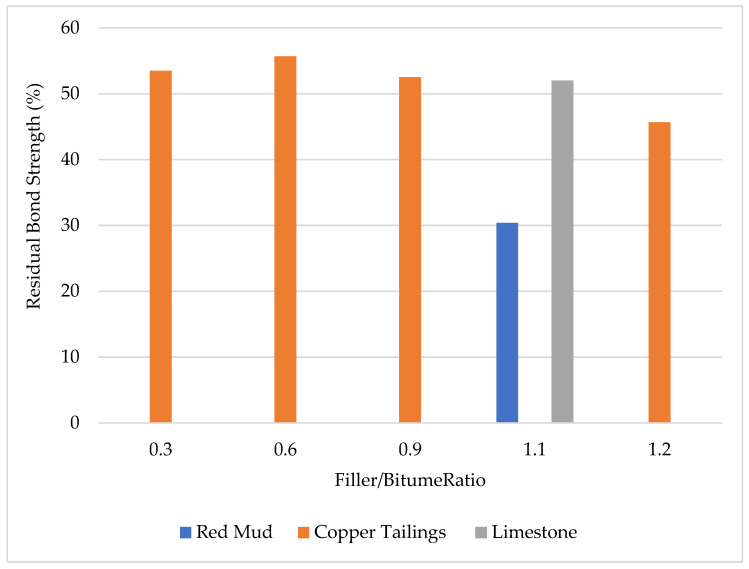
Seven-days residual bond strength of tailings-modified asphalt binder–mastics [40,46,58].

The moisture resistance of mine-tailings modified asphalt mastics compared with limestone mastics obtained from some research is presented in Figure 5. From the figure, it can be inferred that mine tailings had an adverse effect on asphalt binder–mastics moisture resistance [40,58]. The high alkaline metal content in the tailings could be responsible for the poor moisture resistance of the asphalt mastic prepared, by which the bonds formed between bitumen and alkali metals are soluble in the presence of moisture [58]. Additionally, the lack of a sufficient amount of CaO in tailings could also be responsible for the moisture damage. Therefore, it is crucial to improve the moisture susceptibility of mine tailings-modified asphalt mastics through treatment and incorporation anti-stripping additives and hybrid fillers.

### 5.4. Moisture Performance of Mine Tailings-Modified Asphalt Binder–Mastics

Mine tailings–asphalt interaction refers to the way in which mine tailings as fillers interact with asphalt in mastics. This interaction is crucial in determining the properties of mastic, influencing factors like rheological properties, durability, and cost [68]. After incorporating mine tailings in mastic, the interactive ability must be characterized.

The interaction ability of asphalt and filler is commonly measured by K.D. Ziegel−B−G (abbreviated as *K*−*B*−G) techniques (the formulation is shown in Equations (2)–(5)). Stronger asphalt and filler interaction ability is represented by the bigger value of *K*−*B*−G.

(2) $$\frac{G_{c}}{G_{m}}=\frac{1+1.5\varphi }{1-\varphi }$$

(3) $$\frac{G_{c}}{G_{m}}=\frac{1+1.5\varphi B}{1-\varphi B}$$

(4) $$\varphi =\frac{\left(m_{f}/m_{m}\right)}{\left(m_{f}/m_{m}\right)+\rho_{f}/\rho_{m}}$$

(5) $$K-B-G=\left|\frac{\frac{G_{c}}{G_{m}}-1}{\left(1.5+\frac{G_{c}}{G_{m}}\right)\times \varphi }\right|\;$$

where *B* is the interaction degree between the mineral filler and asphalt binder, *G_m_* and *G_c_* are the complex modulus of asphalt binder and the corresponding asphalt mastic, respectively, m_F_ is the weight of filler (*g*), *φ* is the volume fraction of mineral filler, m_m_ is the weight of asphalt binder (*g*), ρ_m_ is the density of asphalt binder (g/cm^3^), and ρ_f_ is the filler density (g/cm^3^).

In [69], the *K*−*B*−G was used to measure the interaction of coal gangue (CG) with asphalt binder and the results were compared with limestone mastic. It was concluded that the CG particles interaction with the Pen70 asphalt binder was slightly lower than that of limestone mastic. Moreover, when the CG was treated with thermal and chemical, the interaction was stronger with the Pen70 asphalt binder compared to limestone. Specifically, the *K*−*B*−G values varied from 0.76 to 1.53 and 0.91 to 1.74 for chemical- and thermal-treated CG, respectively.

**Figure 6:**
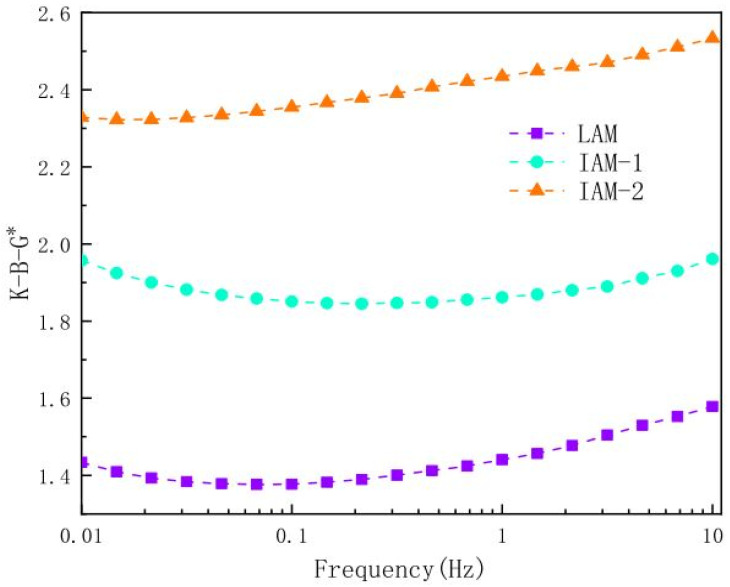
Interaction capacity of iron tailings with asphalt binder [12].

The interaction capacity of iron tailings (ITs) with 70# asphalt binder was investigated by [12]. As shown in Figure 6, it was reported that due to the complex angular properties, microstructure, and particle size distribution of ITs, its K-B-G values were higher than those of limestone. The interactive effect of graphite tailings (GTs) binder using 60/80 asphalt was investigated by [41]. The K-B-G values obtained shows GTs exhibit higher K−B-G values than limestone mastic in the testing temperature range, indicating that GTs have an enhanced asphalt−filler interaction.

Table 5 presents the interactive effect of fillers with bitumen using the K-G-B approach. Based on the results, mine tailings interacted well with asphalt binder. Nonetheless, in some tailings, treatment may be required to enhance their interaction capacity with asphalt binder.

### 5.5. Microstructural Characterization of Mine Tailings-Modified Asphalt Binder–Mastics

**Figure 7:**
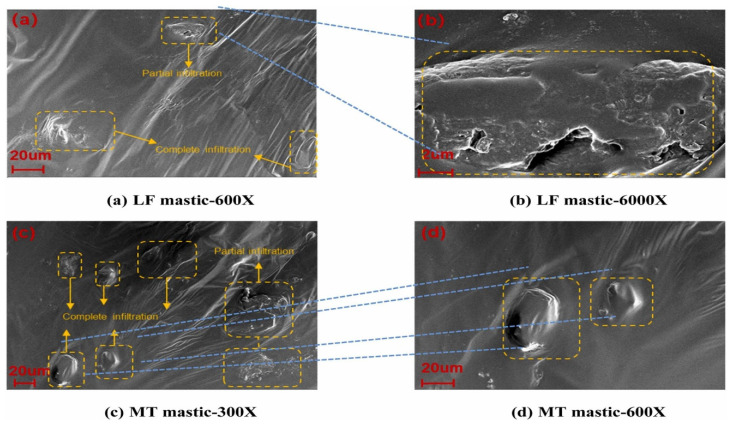
Microstructural characteristics of asphalt binder–mastic with molybdenum tailings (MTs) and limestone filler (LF) [22].

The physical and rheological performance of mine tailings-modified asphalt binder–mastic have demonstrated their effectiveness as sustainable materials. Further insight to the microstructural analysis of these materials in asphalt binder–mastic would help elucidate the bonding mechanisms and give insight into the interactive ability and formation of asphaltene products, enhancing the understanding of the materials’ durability and strength. Using scanning electron microscopy (SEM), ref. [22] evaluated the microstructure characteristics of molybdenum tailings (MT)-modified asphalt binder–mastic. As shown in Figure 7, the surface of MT absorbed more asphalt, resulting in enhanced adhesion between asphalt and MT particles. Also, compared with limestone fillers (LFs), as shown in Figure 7a,b, MT has a greater adhesion with asphalt, which is due to the surface of MTs being rougher than that of LFs. Also, the proportion of MTs to complete permeability in asphalt is more, and the proportion of partial permeability is less in contrast to LFs.

**Figure 8:**
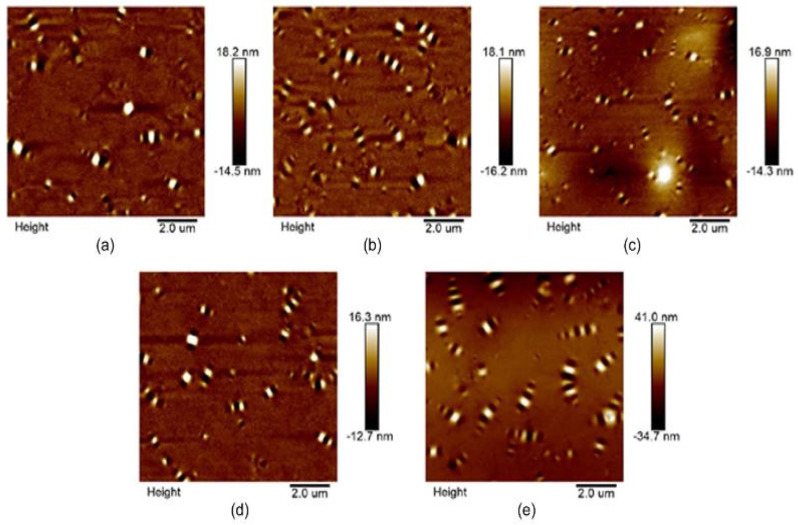
Coal gangue-modified asphalt binder–mastic microscopic characteristic at different filler binder ratios: (**a**) 0.6-; (**b**) 0.9-; (**c**) 1.2-; (**d**) 1.5-; and (**e**) 0.9-aged [70].

The surface morphology of coal gangue-modified asphalt binder–mastic microscopic characteristics was analyzed using atomic force microscopy (AFM) by [70]. As depicted in Figure 8, they observed that the interaction between filler and asphalt varied depending on the filler/binder ratio. Also, they reported that the dispersing effect of the filler was significant at low filler/binder ratio, and the adsorption effect was dominant at high filler/binder ratio. The summary of microstructural characteristics of tailings-modified asphalt binder–mastic is shown in Table 6. It has been generally reported that the physical, chemical, and microstructural features of mine tailings and filler/binder ratio result in variable filler–asphalt interaction, which could provide variable rheological properties to the asphalt binder–mastics. Also, the microstructural analysis reveals that mine tailings adsorbed more asphalt due to their irregular shapes and complex surface geometries. This is the reason for improved asphalt binder–mastic performance. Nonetheless, for better performance, there is need of tailing pretreatment and activation.

### 5.6. Economic and Environmental Impact of Mine Tailings-Modified Asphalt Binder–Mastics

Mine tailings, as a waste material in mining activities, contain different toxic substances and heavy metal elements [71,72,73,74]. When used on pavement, leaching by surface water and groundwater might be the issue [53]. It is essential to investigate the environmental vitality of mine tailings in asphalt mastics in order to promote its environmental friendliness and to ensure their safe use in asphalt pavements. For this reason, heavy metal mobilization is very important in terms of the environmental analysis of tailings in asphalt mastic.

The environmental vitality of tailings in asphalt mastic is usually conducted using toxicity characteristics leaching procedure (TCLP) [75]. The results of TCLP as obtained from the literature are shown in Table 7. It can be seen that toxicity contents of the tailings-modified asphalt mixtures are far below the specified limitations, indicating that these tailings have a lesser pollution risk in short-term leaching and can be utilized in asphalt mixture at a large scale. The lower toxicity content of tailings in asphalt mastics may be due to the encapsulation effect of the bitumen [76], thereby immobilizing the heavy metals present in the tailings. Hence, asphalt mixtures can be used for safe disposal of hazardous materials, like tailings. Nonetheless, since limited studies have been conducted on this subject in the literature, there is need for further studies, especially in terms of long-term leaching effect.

The evaluation of cost effectiveness of incorporating mine tailings in asphalt mastics must be quantified to further reinforce its economic viability. Using the benefit–cost ratio (BCR) and present value (NPV), in Equations (6) and (7) [40], it was evaluated that the productivity of total economic profits of using copper tailings (CT) in asphalt mixtures. The total cost (TC) and total benefit (TB) with consideration of the return by the discount rate (10 years) increased with the increasing of the filler/binder ratio. They concluded that the large-scale utilization of CT can consume an approximate 7.4 million tons of CT that can be recycled in asphalt mixtures yearly and, at the same time, save the consumption of limestone by approximately 7.8 million tons. Using the same parameter of NPV and BCR, ref. [53] also reported that for each kilometer of asphalt pavement construction, iron tailings of 58–115 tons can be consumed which will result in the saving of 50–100 tons of limestone.

(6) $$NPV=\sum_{i}B_{i}{\left(1+r\right)}^{-i}-\sum_{i}C_{i}{\left(1+r\right)}^{-i}=TB-{TC}_{i}=0,1,\ldots ,T\;$$

(7) $$BCR=\frac{\sum_{i}B_{i}{\left(1+r\right)}^{-i}}{\sum_{i}C_{i}{\left(1+r\right)}^{-i}}=\frac{TB}{TC}\;i=0,1,\ldots ,T\;$$

where $B_{i}=\sum_{i}b_{i},$ b_i_ denotes the segments of benefits at *i* year; r is the discount rate;$\;C_{i}=\sum_{i}c_{i},\;$c*_i_* denotes the segments of cost at *i* years; T denotes period time; and TC and TB are the total cost and total benefits in the period, respectively.

Also, ref. [15] conducted cost analysis results from the usage of iron tailings in asphalt pavement by estimating its consumption and the corresponding savings in limestone using 1 km of 2-lane asphalt pavement. It was revealed that the use of IT in place of limestone results in 91.23% reduction in the cost of the asphalt pavement. Ref. [77] conducted the economic viability of red mud in asphalt mixtures. It was observed that as the red mud dosage increases, the total cost (TC), the net present value (NPV), and the total benefit (TB) increases. The BCR is 1.19 and the TB was observed to be greater than 0, which implies that red mud in asphalt mixtures is economically profitable.

**Figure 9:**
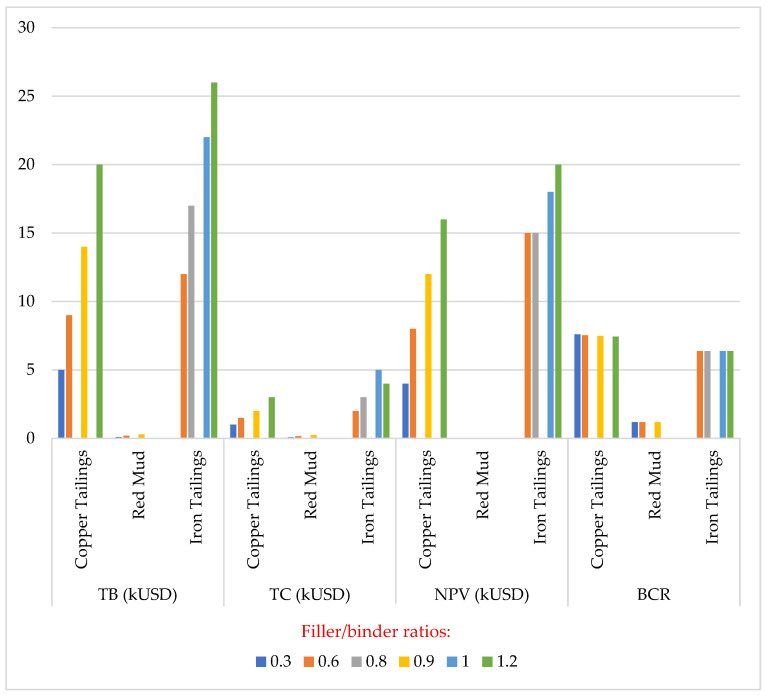
Economic viability of tailings in asphalt mastics from various authors [40,55,77].

Figure 9 depicts the economic viability of tailings in asphalt mastic as reported by various researchers. As seen from the figure, as the filler/binder ratio increases, there is an increment in TC, TB, and NPV. Also, as reported by various authors, no significant change was observed in BCR values. Nonetheless, the BCR values recorded are more than 1, indicating that incorporation of mine tailings in asphalt mastics would result in considerable economic benefits.

Generally, based on environmental impact and economic valuation, the tailings could be considered a potential alternative filler to substitute the traditional fillers. The use of tailings in application can not only reduce the pressure of exploitation of natural resources and protection of urban and rural construction, but it also brings considerable economic profits to enterprises for building, which is beneficial to both sides [40].

## 6. Performance Comparison Between the Tailings as Fillers in Asphalt Binder–Mastic

The performance comparison between the various tailings as fillers in asphalt binder–mastic is depicted in Table 8. Given the variations in asphalt type, filler/binder ratio and mixing conditions, direct comparisons of tailings performance in asphalt binder–mastics are challenging due to inherent discrepancies. These differences may significantly influence the observed performance, rendering the comparisons somewhat inconclusive. Red mud produces the highest complex modulus performance against the other tailings, indicating improved stiffness and resistance to deformation [57]. This can be due to the high SiO₂ and Fe₂O₃ content in the red mud compared to other tailings (see Table 2). Additionally, red mud’s large density allows it to absorb more bitumen, which increases the stiffness and improves the performance of the asphalt binder–mastic. Also, coal gangue and red mud produced a lower phase angle, signifying more elastic mastic behavior. CaO content can influence the phase angle by affecting the mastic’s adhesion and cohesion properties. Therefore, it is desirable to have a higher content of CaO because a lower proportion results in the reduction in adhesion with bitumen [79] and consequently a reduced moisture resistance of the mixture. For conventional properties, asphalt binder–mastics with desirable performance typically exhibit lower penetration values (indicating a harder, stiffer binder), higher softening points (signifying improved resistance to high-temperature deformation), and higher viscosities (indicating enhanced resistance to rutting) [80,81]. The red mud had a softening point and penetration value of 64.2 and 23.9 dmm, respectively, indicating a best performance among the tailings.

In general, the combination and proportion of physical and chemical components in tailings can significantly affect the performance of asphalt binder–mastic [82]. Nonetheless, the effects of these components and their interaction can be complex, as well as with other factors such as asphalt type and filler/binder ratio. Further research is needed to fully understand the relationships between tailings composition and asphalt binder–mastic performance.

## 7. Challenges and Future Directions

The utilization of mine tailings in asphalt binder–mastics results in improved performance. However, there are also limitations and challenges that need to be addressed for future applications.

(1) Potential environmental pollution: The use of mine tailings in asphalt mixtures is generally regarded safe in terms of leaching of heavy metals. However, there is still potential environmental risk if they contain a high proportion of hazardous elements. Hence, there is need for rigorous evaluation and testing to further ensure their environmental friendliness.

(2) Variability in tailings characteristics: The properties of mine tailings vary significantly depending on their source and composition which could affect their performance as asphalt mastics materials. Hence, there is need for careful characterization and selection.

(3) Optimization of tailings content: While mine tailings can significantly influence some properties of asphalt binder–mastics, they negatively affect some other properties. Hence, the optimal content of tailings in asphalt binder–mastics needs to be carefully determined to balance their benefits and potential drawbacks. Furthermore, the development of optimal dosage will ensure industrial scale utilization in asphalt binder–mastics.

(4) Synergistic blends of mine tailings with other materials: the utilization of mine tailings with other materials in asphalt mixtures is yet unknown and should be further looked into. This synergistic blend provides an approach to mitigating the challenges of the mine tailings non-activity, chemical compositions fluctuation, heavy metals immobilization, and their pollution effects [83].

(5) Lifecycle assessment (LCA): Full-scale LCA is advocated to fully understand mine tailings impact in terms of their sustainability and their influence on the environment.

(6) Tailing treatment: Studies have also reported the adverse effect of mine tailings on the moisture susceptibility of asphalt mixtures. Hence, mine tailings need to be activated or pre-treated to improve their efficiency. Generally, the treatment helps improve adhesion, moisture susceptibility and aging resistance along with the low temperature of asphalt binder with inorganic fillers by enhancing the reactivity of mine tailings.

(7) Correlation of tailings composition with asphalt binder–mastic performance: The composition of tailings substantially impacts the performance of asphalt binder–mastic. However, the complex interactions between the tailing components, asphalt type, and filler/binder ratio necessitate further investigation to elucidate the relationships between tailings composition and mastic performance.

## 8. Conclusions

The inclusion of mine tailings in asphalt binder–mastics offers a promising solution for sustainability and cost-effectiveness with enhanced mastic performance. While challenges such as variability in tailings characteristics and potential environmental risks need to be addressed, the overall benefits of using mine tailings in asphalt binder–mastic make them a valuable alternative to traditional materials. The following conclusions are drawn from the study:

- The substitution of traditional filler with mine tailings can significantly reduce the cost of asphalt mixture construction,
- The use of mine tailings enhances the performance of asphalt binder–mastic, leading to extended service life of pavement,
- The utilization of mine tailings as a replacement for conventional fillers will reduce the demand for these non-renewable materials and will help to conserve them while also reducing the environmental impact brought about by their extraction,
- The incorporation of mine tailings in asphalt binder–mastics provides a sustainable solution for their reuse, leading to reduction in the need for landfilling and consequently minimizing the environmental impact by their disposal,
- Studies on mine tailings-modified asphalt binder–mastic have been concentrated on few tailings. The impact of other tailings such as gold tailings should also be investigated,
- In evaluating the environmental impact of mine tailings in asphalt binder–mastic, much focus has been given to analyzing the leaching of heavy metals and other environmental concerns such as VOC and PAH emissions associated with asphalt mixtures have been overlooked,
- The complex interactions between the tailing components, asphalt type, and filler/binder ratio necessitate further investigation to elucidate the relationships between tailings composition and mastic performance.

The utilization of mine tailings as a valuable component in asphalt binder–mastic not only enhances sustainability in the pavement industry but also promotes circular economy principles by transforming waste materials into valuable resources.

## Figures and Tables

**Table 4:** Effects of tailings on the performance of asphalt binder–mastics.

Tailings	Bitumen	Achievement	Filler/Binder Dosage	Reference
Graphite Tailings (GTs)	60/80	GTs improved the high-temperature rutting resistance by 14.73%, fatigue damage resistance by 225%, and had a 20.42% improvement in low-temperature crack resistance.	50/50	[41]
Copper Tailings (CTs)	60/80	The stiffness of asphalt mastics with CTP was improved by 35~65% compared to LP.	0.3, 0.6, 0.9, 1.2	[40]
Molybdenum Tailings (MTs)	80/100	MTs-modified asphalt mastic has improved rut resistance of approximately 1.08–1.22 times over limestone mastic.	0.3, 0.6, 0.9, 1.2	[22]
Iron Tailings (ITs)	60/80	ITs enhance the stiffness of mastics by 30–60%.	0.6, 0.8, 1.0, 1.2	[55]
Coal Gauge (CG)	60/–80	The mastics containing CG have a higher softening point, smaller penetration, and improved temperature stability.	0.6, 0.9, 1.2, 1.5	[56]
Magnetite	C170 and C320	Magnetite fillers in asphalt binder–mastics showed enhanced rheological behavior compared to natural limestone filler.	0.5, 1.0 and 1.5	[23]
Activated Coal Gauge (ACG)	100/120	The ACG improved the asphalt mastic’s temperature sensitivity and shear strength better than conventional limestone filler.	1:1	[45]
Red Mud	60/80	Red mud-modified mastic resulted in a better stiffness and elastic recovery than the limestone.	1:1	[33,57,58]

**Table 5:** Interactive effect of fillers with bitumen using the K-G-B approach.

Study	Filler	Bitumen	Replacement Level	Findings
[15]	Iron tailings	60/80	0-100% with 25% increment.	Iron tailings had a negative effect on the interaction between the asphalt binder and filler.
[12]	Iron tailings	60/80	100%	The K-G-B of iron tailings-modified mixture is 32% and 70% higher than limestone-modified mixture.
[41]	Graphite tailings	60/80	NA	Graphite tailings have a stronger asphalt–filler interaction of about 16%, 19%, and 35% more than the limestone fillers.
[69]	Coal gangue	60/80	100%	The interaction of coal gangue with asphalt was improved and significantly higher than limestone when the coal gangue was treated thermally and chemically.

**Table 6:** Microstructural characteristics of tailings-modified asphalt binder–mastic.

Tailings	Bitumen	Microstructural Method	Findings	Reference
Coal Gangue (CG)	60/80	AFM	CG has the largest proportion of number and area ratio of bee-type structures compared to limestone and cement, indicating that CG has a stronger interaction with emulsified asphalt.	[56]
Molybdenum Tailings	90#	SEM	Molybdenum tailings adsorbed more asphalt due to their irregular shapes and complex surface geometries.	[22]
Graphite Tailings	60/80	AFM	A strong asphalt–filler interaction was observed in graphite tailings (GTs)-modified asphalt binder–mastic. Also, GT filler absorbed wax and asphaltene, creating a thick layer of structural asphalt.	[41]
Iron Tailings	70#.	SEM	Due to the smaller particle size of Iron tailings (ITs), ITs-modified asphalt binder–mastic contains more particles than in limestone mastics, aiding ITs in adsorbing more asphalt on its particle surface.	[55]
Magnetite Tailings	C170	SEM	It was observed though that no major clusters of magnetite particles were formed in the mastic.	[23]

**Table 7:** Heavy metal elements concentration in tailings.

Tailings	Toxic Metal Elements (ug/L)						Ref.
	Cr	Cu	Pb	Zn	Cd	Ba	
Copper	8	82	7	30	-	-	[40]
Iron	2.28	26.77	0.20	105.79	0.27	61.61	[53]
Red mud	261	297	638	1010	-	184	[77]
Toxicity threshold (ug/L)	15,000	100,000	5000	100,000	1500	100,000	[78]

**Table 8:** Comparative performance of tailing types in asphalt binder–mastic.

Tailings	Blending Conditions	Bitumen	Penetration (dmm)	Softening Point (°C)	Ductility (mm)	Viscosity (Pa.s)	Phase Angle (°)	Complex Modulus (kPa)	Ref.
Red mud	Temp.: 150 °C; f/b ratio: 1.1; speed: 1000 rpm for 30 min.	60/80	23.9	64.2	30.5	4.2	77.5	100	[57]
Magnetite	Temp.: 150 ± 5 °C; f/b ratio: 0.5–1.5; speed: 2000 rpm for 6 min.	C170	-	-	-	80	-	75	[23]
Copper	Temp.: 150 ± 5 °C; f/b ratio: 0.3–1.2; speed: 1000 and 300 rpm for 30 and 10 min, respectively.	AH70	31.8	60.3	47.7	-	79.5	68	[40]
Iron	Temp.: 150 ± 5 °C; f/b ratio: 0.6–1.2; speed: 500 and 1000 rpm for 5 and 30 min, respectively.	60/80	-	-	-	7.2	78	84	[55]
Coal gangue	Temp.: 140 °C; f/b ratio: 1.0; speed: 500 and 3000 rpm for 5 and 30 min, respectively.	60/80	30	20.5	20.5	-	68.5	50	[62]
Molybdenum	Temp. 140 °C; f/b ratio: 0.3–1.2; mixing time: 10 min.	90#	-	-	-	3.76	81.1	16.36	[22]

## Data Availability

No new data were created or analyzed in this study. Data sharing is not applicable to this article.
